# Predictors of modern contraceptive methods use among married women of reproductive age groups in Western Ethiopia: a community based cross-sectional study

**DOI:** 10.1186/s12905-015-0208-z

**Published:** 2015-07-17

**Authors:** Tesfalidet Tekelab, Alemu Sufa Melka, Desalegn Wirtu

**Affiliations:** Lecturer of Reproductive and Maternal Health, Department of Nursing and Midwifery, College of Medical and Health Sciences, Wollega University, Nekemte, Ethiopia; Lecturer of Reproductive Health, Department of Public Health, College of Medical and Health Sciences, Wollega University, Nekemte, Ethiopia; Assistant Professor of Reproductive Health, Department of Public Health, College of Medical and Health Sciences, Wollega University, Nekemte, Ethiopia

**Keywords:** Modern contraceptive, Associated factors, Nekemte town

## Abstract

**Background:**

In Ethiopia, the prevalence of modern contraceptive use is very low (27 %) and the percentage of those with unmet needs for family planning is 25 %. The current study identified factors associated with the utilization of modern contraceptive methods among married women in Western Ethiopia.

**Methods:**

A community based, cross-sectional study was employed from April 10 to April 25, 2014, among married women of reproductive age in Nekemte Town. A multi-stage sampling procedure was used to select 1003 study participants. A pretested structured questionnaire was used to collect data, and data collectors who had completed high school were involved in the data collection process. A bivariate, multivariable logistic regression model was fit, and statistical significance was determined with a 95 % confidence level.

**Result:**

The overall utilization rate of modern contraceptives in this study was 71.9 %. The most common form of modern contraceptives used was injectable (60.3 %). Age (AOR = 2.00, 95 % CI = 1.35–2.98), women’s educational level (AOR = 2.50, 95 % CI = 1.62–3.84), monthly income (AOR = 2.26, 95 % CI = 1.24–4.10), respondent’s fertility (AOR = 2.60, 95 % CI = 1.48–4.56), fertility-related decision (AOR = 3.70, 95 % CI = 2.45–5.58), and having radio (AOR = 1.93, 95 % CI = 1.37–2.71) showed significant positive associations with the utilization of modern contraceptive methods.

**Conclusions:**

The findings showed that women’s empowerment, fertility-related discussions among couples, and the availability of the media were important factors that influenced the use of modern contraceptives. Thus, policymakers and implementers should work on those factors to increase the utilization of modern contraceptive methods.

## Background

The widespread adoption of family planning (FP) represents one of the most dramatic changes of the 20^th^ century. The growing use of contraception around the world has given couples the ability to choose the number and spacing of their children and has had great lifesaving benefits. Despite these impressive gains, contraceptive use is still low and the need for contraception is high in some of the world’s poorest and most populous places [1].

Full access to reproductive health care is crucial to attaining many of the Millennium Development Goals (MDGs). Increasing contraceptive prevalence and reducing unmet need for family planning are indicators of progress toward Goal 4 and 5, improving maternal and child health [2].

Women’s ability to space and/or limit pregnancies has a direct impact on their health and well-being as well as on the outcome of each pregnancy. Family planning is a major contributing factor towards child survival and reduction in maternal mortality. The relevance of FP in any strategy for safe motherhood and child survival is undeniable [1, 3].

Family planning can reduce the number of deaths among women by preventing unintended pregnancies, which account for about 30 % of all births in sub-Saharan Africa [4, 5]. In 2008, annual rates of unintended pregnancies were highest in Africa, at 86 per 1000 women aged 15–44 years [6].

According to the 2011 Ethiopia Demographic and Health Survey (EDHS) report, 27 % of married Ethiopian women of childbearing age (15–49) use modern method of family planning; this is a dramatic increase from 2005 when only 14 % of married women of childbearing age were using any modern contraception. However, 25 % of the surveyed married women either do not want any more children or they want to wait for two or more years before having another child. Despite this, the women were not using any form of contraception [7].

Previous studies have shown that factors such as maternal age, parity, maternal and husband educational attainment, place of residence, and decision making in the household were associated with the use of modern contraceptive methods [8–11]. This study identified factors associated with the utilization of modern contraceptive methods, which helps policy makers and health managers to design effective strategies based on the findings.

## Methods

### Study design, setting and participants

A community-based cross- sectional study design was carried out from April 10 to April 25, 2014, among married women of reproductive age in the Town of Nekemte, Oromia Region, West Ethiopia. Nekemte Town is a capital of East Wollega Zone (Province) located at 321 KM from Addis Ababa to the west. The total population of the town is estimated to be 75,219 of which 38,385 (51 %) were females [12]. In the town there are one public hospital and two health centers and five NGOs working in the area of reproductive health including family planning. On top of this there are different categories of health professionals in the town who are working on family planning. All form of modern contraceptive methods are available free of charge. All the participants of the study were married women, ranging from 15 to 49 years in age, and they have lived in the study area for at least 6 months. Women who were critically ill, could not provide informed consent, pregnant women or infecund were excluded from the study.

### Sample size and sampling techniques

The sample size was determined using a formula for estimation of single population proportion with the assumption of 95 % confidence interval, a margin of error of 4 % and taking 25.4 % contraceptive prevalence of Butajira district,Ethiopia [11] and a design effect of 2. To avoid the effect of the design that decreases the representativeness of the study we used design effect. To compensate the non-response rate, 10 % of the determined sample was added up on the calculated sample size and the final sample size was 1004.

A multi-stage sampling technique was employed for the selection of the sampling units. First, three sub-cities were selected from six sub-cities found in Nekemte Town. Next, four zones were selected from each sub-city randomly. Then the calculated sample sizes were proportionally allocated to each zone based on the number of married women living in each zone. Then picking a house randomly for the initial household from each zone, the final households with married women were selected using systematic sampling from the existing sampling frame of households. Finally, eligible married women of reproductive age group were interviewed from each selected households. When two or more married women are there in a household, only one woman was randomly selected for an interview in order to avoid intra-class correlation.

### Data collection procedures

Pre-tested structured questionnaires were adapted from different literature. The questionnaires were prepared in English, translated into Afan Oromo (regional language), and then retranslated back to English by people who are proficient in both languages to maintain the consistency of the questionnaires. To administer the structured questionnaires, five female high school graduates were selected from the study area. Training was given for three days about the objective, relevance of the study, confidentiality of information, respondent’s rights, informed consent and techniques of interview. Moreover, a practical demonstration of the interview was carried out in the classroom. Four supervisors who have second degree oversaw the data collection procedures. All field questionnaires were reviewed each night and issues that arose during data collection were addressed in morning sessions.

### Data processing and analysis

Data were cleaned and entered into a computer using Epi-Info window version 6.5 statistical programs. The data were then exported to SPSS windows version 20.0 for further analysis. The descriptive analyses such as proportions, percentages, frequency distribution and measures of central tendency were conducted.

Initially, bivariate analysis was performed between dependent variable and each of the independent variables, one at a time. Their odds ratios (OR) at 95 % confidence intervals (CI) and *p*-values were obtained. The findings at this stage helped us to identify important associations. Then all variables found to be significant at bivariate level (at *p* <0.05) were entered into multivariate analysis using the logistic regression model to test the significance of the association.

### Operational definition

- **Modern contraceptive Methods** - female sterilization, male sterilization, the pill, the intrauterine device (IUD), injectables, implants, condom and diaphragm/foam/jelly.
- **Traditional methods** - rhythm (Calendar method), withdrawal and folk.

### Ethical considerations

Ethical clearance and permission was obtained from Wollega University Institutional Review Board. Permission was secured from all sub-cities of Nekemte Town through a formal letter. Written informed consent was obtained from each respondent before their interview. Confidentiality of individual client information was ensured by using unique identifiers for the study participants and also limiting access to respondents’ information to the principal investigator and research assistants by storing the completed questionnaires and all documents with participant information in a lockable cabinet.

## Results

1. Socio-demographic characteristics

A total of 1003 married women of reproductive age responded to the questionnaire, making a response rate of 99.9 %. Half of the respondents (51.6 %) were in the age group of 25–34 years with median age of 28 years. Three fourths of the respondents (74.8 %) were from Oromo ethnic group. About half of the respondents (48.4 %) were protestant in religion. Two hundred ninety-one (27.9 %) respondents had completed secondary level education, whereas 29.4 % of the respondents’ husbands had attended college and above. More than half of the respondents (52.9 %) and their husbands (42.7 %) were housewives and daily laborers, respectively. Their mean monthly income was 1510.6 Ethiopian birr (ETB). Out of the total married women, 72.1 % owned radio/TV (Table 1).

**Table 1:** Socio demographic characteristics of Married women in Nekemte town, Ethiopia, April, 2014

Variables (1003)	Number (%)
Age category	
15–24	281 (28.0)
25–34	514 (51.2)
35–44	198 (19.7)
> 44	10 (1.0)
Ethnicity	
Oromo	750 (74.8)
Amhara	200 (19.9)
Tigre	36 (3.6)
Others*	17 (1.7)
Religion	
Protestant	485 (48.4)
Ethiopian Orthodox	423 (42.2)
Catholic	17 (1.7)
Muslim	76 (7.6)
Others**	2 (0.2)
Educational status of the respondent	
Can’t read and write	113 (11.3)
Can read and write	71 (7.1)
Grade 1–4	142 (14.2)
Grade 5–8	206 (20.5)
Secondary	280 (27.9)
College and above	191 (19.0)
Educational status of the husband	
Can’t read and write	59 (5.9)
Can read and write	48 (4.8)
Grade 1–4	81 (8.1)
Grade 5–8	249 (24.8)
Secondary	271 (27.0)
College and above	295 (29.4)
Occupational status of the respondents	
Governmental Employee	157 (15.7)
Daily laborer	157 (15.7)
Housewife	531 (52.9)
Merchant	99 (9.9)
Student	53 (5.3)
Others	6 (0.6)
Occupational status of the Husband	
Governmental Employee	359 (35.8)
Daily laborer	428 (42.7)
Merchant	153 (15.3)
Student	19 (1.9)
Others****	44 (4.4)
Income (ETB)	
< 600	221 (22.0)
600–1000	275 (27.4)
1001–1500	135 (13.5)
1501–2000	179 (17.8)
> 2000	193 (19.2)
Mean	1533 ETB
Have radio/TV	
Yes	723 (73.1)
No	280 (27.9)

Other* = Gurage, Shinasha, other** = Wakefeta, Jehovah’s Witness, other*** = petty maker, house maid, other**** = Driver, carpenter 1$ = 20ETB

2. Fertility and reproduction related characteristics

Majority of the respondents (95.8 %) had been pregnant at least once during their lives and mean number of living child was 2.4. Three hundred sixty-three (37.8 %) of the study participants had less than or equal to two living children. More than half (53.2 %) of the study participants expressed future desire for more children. Out of those who desired to have children, (62.9 %) desired to have one or two children. From those who desired to have children in the future, 70.4 % of the respondents expressed a desire to bear more children in order to have a bigger family. Half of the respondents’ partners (50.3 %) desired to have children in the future. A majority of the respondent (84.8 %) decided on fertility issues jointly with their partners (Table 2)

**Table 2:** Fertility desire and reproductive history of married women in Nekemte town, Ethiopia, April, 2014

Variables	Number (%)
Have you ever pregnant (1003)	
Yes	961 (95.8)
No	42 (4.2)
Number of children alive (961)	
= <2	598 (62.2)
> 2	363 (37.8)
Future fertility desire (1003)	
Yes	534 (53.2)
No	398 (39.7)
I don’t know	71 (7.1)
Number of desired child (385)	
1–2	242 (62.9)
> 2	143 (37.1)
Reason for future child desire (534)	
Have few children	376 (70.4)
Need of son	102 (19.1)
Death of child	20 (3.8)
No response	48 (9.0)
Other*	6 (1.1)
Partner fertility desire (1003)	
Yes	505 (50.3)
No	370 (36.9)
Don’t know	128 (12.8)
Decision on fertility (1003)	
Wife	36 (3.6)
Husband	116 (11.6)
Jointly	851 (84.8)

Other* = Husband desire, Parent influence

3. Awareness, ever use and current use of modern contraceptive

**Fig. 1:**
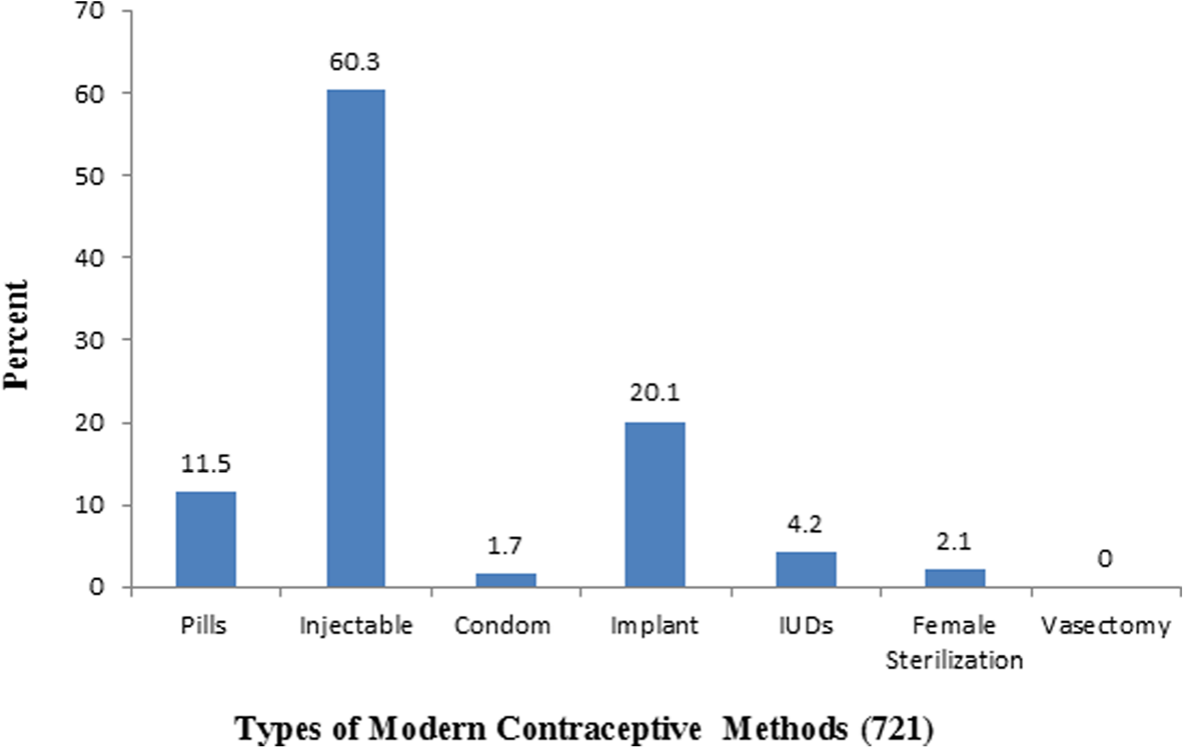
Bar graph showing proportion of respondent’s practicing modern contraceptive methods by type in Nekemte town, Ethiopia, April, 2014

**Fig. 2:**
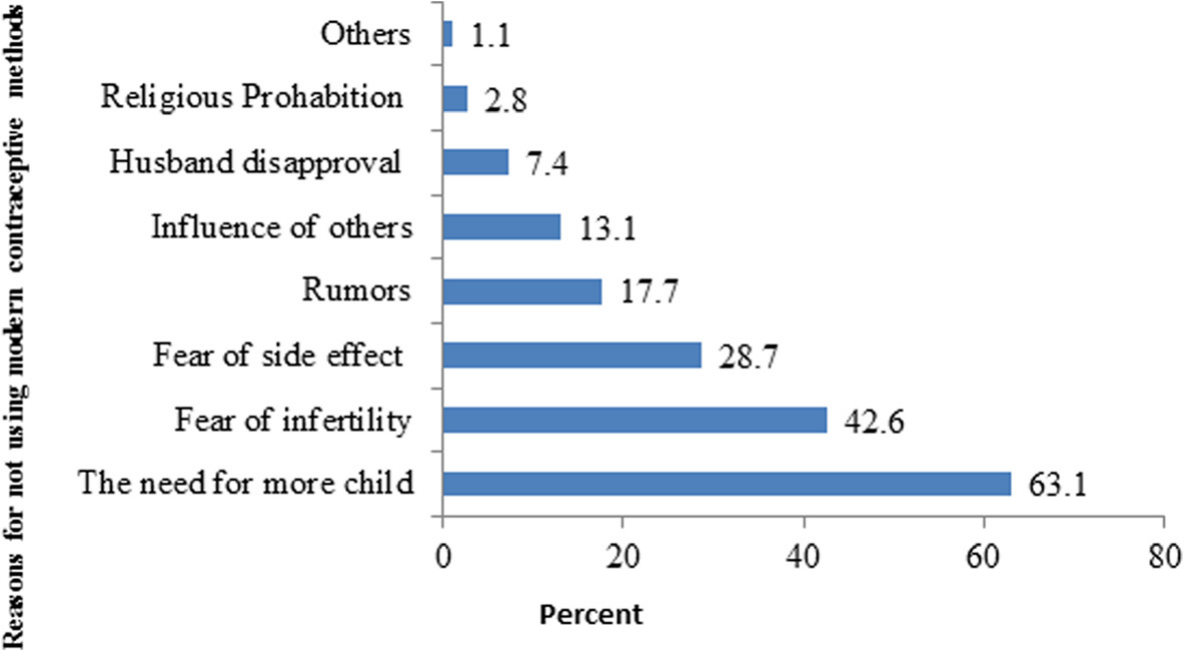
Bar graphs showing reasons for not using of modern contraceptive methods among currently married women in Nekemte town, Ethiopia, April 2014

Knowledge of at least one form of modern contraceptive method was universal (99.9 %). The major source for information regarding modern contraceptive methods was health workers (82.1 %). Injectable form of contraceptive was heard of by 90.7 % of the respondents. Ever use and current use of modern contraceptive were 82 and 71.9 %, respectively (Table 3). In this study, the majority of the respondents used injectable (60.3 %), followed by implant (20.1 %) methods of contraceptives (Fig 1). Various reasons were given during the interviews for not using modern contraception. The main reasons reported by women who were not using contraceptives were the desire for more children, the fear of infertility and the fear of side effects (Fig 2).

**Table 3:** Awareness and use of modern contraceptive methods of married women in Nekemte town, Ethiopia, April, 2014

Variables	Number (%)
Ever heard of modern methods (1003)	
Yes	1002 (99.9)
No	1 (0.1)
Source of information on Modern contraceptive Methods (1002)	
Health worker	823 (82.1)
Radio	739 (73.7)
TV	696 (69.4)
Friends	201 (20.0)
Other*	18 (1.8)
Type of Modern contraceptive Methods ever Heard (1002)	
Pills	909 (90.6)
Injectable	910 (90.7)
Condom	582 (58.0)
IUCD	773 (77.1)
Implant	778 (77.6)
Female sterilization	315 (31.4)
Vasectomy	96 (9.6)
Ever use of Modern contraceptive methods (1003)	
Yes	822 (82.0)
No	181 (18.0)
Current use of modern contraceptive Methods (1003)	
Yes	721 (71.9)
No	282 (28.1)

Other* = newspaper, husband

4. Factors associated with current utilization of modern contraceptive methods

A multivariable analysis was performed to identify independent predictors of utilization of modern contraceptive methods. Age, women’s education, monthly income, respondents’ fertility desire, fertility related decisions and possession of radio showed significant association with utilization of modern contraceptive methods (Table 4).

**Table 4:** A multivariate Logistic regression on predictors of use of modern contraceptive methods in Nekemte town, Ethiopia, April, 2014

Characteristics	Using Modern Contraceptive methods	Crude OR	Adjusted OR
	Yes (%) No (%)	OR (CI)	OR (CI)
Age category in years			
15–24	164 (58.4 %) 117 (41.6 %)	1	1
25–34	399 (77.6 %) 115 (22.4 %)	2.48 (1.81–3.39)	2.00 (1.35–2.98)*
35–44	152 (76.8 %) 46 (23.2 %)	2.36 (1.57–3.53)	1.21 (0.68–2.14)
> 44	6 (60.0 %) 4 (40.0 %)	1.07 (0.30–3.88)	0.59 (0.12–2.82)
Education of respondents			
Below and Primary	330 (62.0 %) 202 (38.0 %)	1	1
Secondary and above	391 (83.0 %) 80 (17.0 %)	2.99 (2.22–4.03)	2.50 (1.62–3.84)*
Education of Husband			
Below and Primary	266 (60.9 %) 171 (39.1 %)	1	1
Secondary and above	455 (80.4 %) 111 (19.6 %)	2.64 (1.99–3.50)	1.21 (0.83–1.78)
Occupation of respondents			
Government Employed	131 (83.4 %) 26 (16.6 %)	2.19 (1.50–3.41)	0.58 (0.32–1.03)
Others	590 (69.7 %) 256 (30.3 %)	1	1
Monthly income			
< 600	132 (59.7 %) 89 (40.3 %)	1	1
600–1000	184 (66.9 %) 91 (33.1 %)	1.36 (0.94–1.97)	1.50 (0.97–2.32)
1001–1500	108 (80.0 %) 27 (20.0 %)	2.70 (1.64–4.45)	2.26 (1.24–4.10)*
1501–2000	137 (76.5 %) 42 (23.5 %)	2.20 (1.42–3.41)	1.29 (0.76–2.21)
> 2000	160 (82.9 %) 33 (17.1 %)	3.27 (2.06–5.19)	1.55 (0.85–2.82)
Number of live children			
< =2	416 (69.6 %) 182 (30.4 %)	1	1
> 2	284 (78.2 %) 79 (21.8 %)	1.57 (1.16–2.13)	0.88 (0.57–1.37)
Respondent Wants more child			
Yes	345 (64.6 %) 189 (35.4 %)	1	1
No	376 (80.2 %) 93 (19.8 %)	2.22 (1.66–2.95)	2.60 (1.48–4.56)*
Husband desire more child			
Yes	331 (65.5 %) 174 (34.5 %)	1	1
No	390 (78.3 %) 108 (21.7 %)	1.90 (1.43–2.52)	1.05 (0.63–1.74)
Fertility related decision			
Joint decision	649 (76.3 %) 202 (23.7 %)	3.57 (2.50–5.09)	3.70 (2.45–5.58)*
Others	72 (47.4 %) 80 (52.6 %)	1	1
Have Radio/Tv			
Yes	557 (77.0 %) 166 (23.0 %)	2.37 (1.77–3.19)	1.93 (1.37–2.71)*
No	164 (58.6 %) 116 (41.4 %)	1	1

Key = *Statistically significant (*p*-value <0.05) 1 = Reference category

Women within the age range of 25–34 years were twice as likely as other age groups to use modern contraceptive methods (AOR = 2.00, 95 % CI: 1.35–2.98). Those respondents who had secondary school education and above were more likely to utilize modern contraceptives when compared to those who had primary school education and below (AOR = 2.50, 95 % CI: 1.62–3.84). Those respondents who earn 1001 to 1500ETB per month were more likely to engage in modern contraceptive methods than others (AOR = 2.26, 95 % CI: 1.24–4.10). Women who do not desire more children in the future were 2.6 times more likely to use modern contraceptives than those who desire children in the future (AOR = 2.60, 95 % CI: 1.48–4.56).

Women who made joint decisions about fertility issues with their husbands were 3.7 times more likely to use modern contraceptives than those who did not make joint decisions (AOR = 3.70, 95 % CI: 2.45–5.58). Respondents who have radio were nearly twice as likely to use modern contraceptive methods as those who had no radio (AOR = 1.93, 95 % CI: 1.37–2.71).

## Discussion

This study identified predictors of current utilization of modern contraceptive methods in Nekemte Town. The overall utilization of modern contraceptives in this study was found to be 71.9 %. The finding was much higher than previous studies in Ethiopia and other developing countries [13–15]. The contraceptive prevalence rate (CPR) of this study was also higher than the urban CPR of 2011 EDHS report which was 50 % [7]. The current finding was similar to a study done in Northern Ethiopia (71.20 %) [16]. The difference could be due to promotion of modern contraceptive methods by health extension workers, availability of range of method choice and facilities providing the service.

The most common form of modern contraceptives used was injectable (60.3 %) followed by implant (20.1 %) and pills (11.5 %). The finding was similar to previous studies done in Ethiopia [11, 13]. The most common modern contraceptive method used by married women in Ethiopia according to EDHS 2011 was injectable [7].

In this study, participants whose age category was 25–34 years were more likely to utilize modern contraceptive methods than other age groups. It is consistent with the studies conducted in Mali, Pakistan and Bangladesh [8, 9, 17]. According to EDHS 2011, the proportion of married women who were using modern contraceptive methods increases until it peaks at 29 % in the 30–34 age groups. Current contraceptive use is lower among young women and among older women (some of whom are no longer fecund) than among those at the intermediate age groups [7].

Educational attainment was an important predictor of modern contraceptive use. In this study, women that had secondary education and above were 2.5 times more likely to use modern contraceptives than those with primary or lower education. This agrees with the studies conducted in Ethiopia and Bangladesh [10, 11, 17, 18].

Twenty-two percent of women with no education report current use of any method, compared with 68 % of women with more than secondary education in Ethiopia [7]. Women’s education, particularly secondary and tertiary education, contribute to women’s empowerment and decision-making regarding fertility related issues and can help them to exercise reproductive health rights.

Women who earn 1001 to 1500 ETB per month were more likely to practice modern contraceptive methods than others. This study was consistent with studies conducted in other developing countries [19]. Inconsistent with the studies conducted in Tanzania and Pakistan [10, 20]. This could be because as an income increase, exposure to different information and financial accessibility of services will be improved.

In this study, those respondents who did not express future desire for children were 2.6 times more likely to utilize modern contraceptives during the study period. The finding was consistent with previous studies conducted elsewhere [13]. It was obvious that women who desire children were not ready to use contraceptives.

In the current study those women who involve their husbands in fertility related decisions were 3.7 times more likely to use modern contraceptives compared to other women. The finding was in line with findings from Zambia [21]. Women who have a role in household and family decisions exercise greater control over their own lives and surroundings. Modern contraceptive use increases with the number of decisions women make jointly with their husbands [22]. The finding magnifies that discussion between partners about fertility issues was an important factor in family planning. Therefore, policy makers and program implementers should consider the importance of joint decision on matters related to reproductive health and design appropriate strategies to encourage couple discussion.

This study indicated that the participants of the study who owned radio/TV were nearly twice as likely to use modern contraceptives compared to their counterparts. Radio is the second most common source of family planning messages at 34 % according to EDHS 2011 for Ethiopian women. The proportion of contraceptive use was also higher for those women who watched television frequently [7, 23].

The drawback of this study was the cross-sectional nature of the data that could obscure the causal effect relationships of different factors. The strength of this study was the large sample size used, which would more represent the source population.

## Conclusions

The overall utilization of modern contraceptives in this study was 71.9 %. The common modern contraceptive method used was injectable (60.3 %). The finding of this study highlighted that women’s empowerment, fertility-related discussions among couples, and the availability of the media were important factors that influenced the use of modern contraceptive methods. Radio/TV programs should emphasize health information about modern contraceptive methods and the various types of contraceptive methods available. Policy makers and implementers should work on those factors to increase the utilization of modern contraceptive methods.

## Abbreviations

EDHS: Ethiopian Demographic and Health Survey
FP: Family planning
IUCD: Intrauterine contraceptive device
MDG: Millennium development goal
